# Influence of Cation Transporters (OCTs and MATEs) on the Renal and Hepatobiliary Disposition of [^11^C]Metoclopramide in Mice

**DOI:** 10.1007/s11095-021-03002-2

**Published:** 2021-02-08

**Authors:** Irene Hernández-Lozano, Severin Mairinger, Michael Sauberer, Johann Stanek, Thomas Filip, Thomas Wanek, Giuliano Ciarimboli, Nicolas Tournier, Oliver Langer

**Affiliations:** 1 Department of Clinical Pharmacology, Medical University of Vienna, A-1090 Vienna, Austria; 2 Preclinical Molecular Imaging, AIT Austrian Institute of Technology GmbH, Seibersdorf, Austria; 3 Medicine Clinic D. Experimental Nephrology, University Hospital Münster, Münster, Germany; 4 Laboratoire d’Imagerie Biomédicale Multimodale (BioMaps), CEA, CNRS, Inserm, Service Hospitalier Frédéric Joliot, Université Paris-Saclay, Orsay, France; 5 Department of Biomedical Imaging and Image-guided Therapy, Division of Nuclear Medicine, Medical University of Vienna, Vienna, Austria

**Keywords:** [^11^C]metoclopramide, CYP2D6, multidrug and toxin extrusion proteins (MATEs), organic cation transporters (OCTs), positron emission tomography (PET)

## Abstract

**Purpose:**

To investigate the role of cation transporters (OCTs, MATEs) in the renal and hepatic disposition of the radiolabeled antiemetic drug [^11^C]metoclopramide in mice with PET.

**Methods:**

PET was performed in wild-type mice after administration of an intravenous microdose (<1 μg) of [^11^C]metoclopramide without and with co-administration of either unlabeled metoclopramide (5 or 10 mg/kg) or the prototypical cation transporter inhibitors cimetidine (150 mg/kg) or sulpiride (25 mg/kg). [^11^C]Metoclopramide PET was also performed in wild-type and *Slc22a1/2*^*(−/−)*^ mice. Radiolabeled metabolites were measured at 15 min after radiotracer injection and PET data were corrected for radiolabeled metabolites.

**Results:**

[^11^C]Metoclopramide was highly metabolized and [^11^C]metoclopramide-derived radioactivity was excreted into the urine. The different investigated treatments decreased (~2.5-fold) the uptake of [^11^C]metoclopramide from plasma into the kidney and liver, inhibited metabolism and decreased (up to 3.8-fold) urinary excretion, which resulted in increased plasma concentrations of [^11^C]metoclopramide. Kidney and liver uptake were moderately (~1.3-fold) reduced in *Slc22a1/2*^*(−/−)*^ mice.

**Conclusions:**

Our results suggest a contribution of OCT1/2 to the kidney and liver uptake and of MATEs to the urinary excretion of [^11^C]metoclopramide in mice. Cation transporters may contribute, next to variability in the activity of metabolizing enzymes, to variability in metoclopramide pharmacokinetics and side effects.

## Introduction

Metoclopramide is a gastroprokinetic and antiemetic drug commonly prescribed for the treatment and prevention of various gastrointestinal disorders such as gastroparesis, esophageal reflux, dyspepsia, or chemotherapy-related nausea (1). In 2009, the FDA issued a black box warning regarding the use of metoclopramide because of its side effects in the central nervous system (2). Central side effects associated with long-term treatment with metoclopramide include tremors and Parkinson-like symptoms, which are caused by blockade of dopamine D_2_ receptors in the basal ganglia (3). Moreover, it has been reported that after prolonged or high-dose treatment with metoclopramide patients are at higher risk of developing tardive dyskinesia, a potentially irreversible serious movement disorder (4, 5). In addition, metoclopramide is associated with highly variable clinical response (6).

After oral dosing, metoclopramide undergoes significant first-pass metabolism in the liver, resulting in highly variable oral bioavailability. Approximately 20% of a single oral or intravenous (i.v.) dose of metoclopramide is excreted unchanged in urine, and up to 60% in the form of metabolites (7–9). One major metabolic pathway of metoclopramide is oxidation by CYP2D6 in the liver followed by conjugation (10, 11). An *N*-O-glucuronide has been identified as the major metoclopramide metabolite in human urine and plasma (12, 13). Previous studies have provided evidence for non-linear pharmacokinetics of metoclopramide in rats and humans, suggesting a role of saturable mechanisms in its clearance (9, 14–16). Another study reported dose dependency in the uptake rate constant of metoclopramide from plasma into the rat liver, pointing to the involvement of hepatic uptake transporters in the metabolic clearance of metoclopramide (17).

At physiological pH, more than 99% of metoclopramide is in protonated form, which suggests that cation transporters may contribute to its passage over biological membranes (p*K*a = 9.71, log *D* octanol/water pH 7.4 = 0.46) (18). Metoclopramide is a weak substrate of human and murine P-glycoprotein (P-gp/*ABCB1*) (19, 20), which was shown to mediate in vivo its elimination from the brain in rats, non-human primates and humans (21–23). In vitro studies showed that metoclopramide is also a substrate of human organic cation transporters (OCTs) 1 and 2 (OCT1/*SLC22A1* and OCT2/*SLC22A2*) (24). In humans, OCT1 is predominantly expressed in the basolateral (blood-facing) membrane of hepatocytes and OCT2 in the basolateral membrane of kidney proximal tubule cells, while in mice both OCT1 and OCT2 are expressed in the kidneys (25–27). Due to the functional coupling between OCTs and multidrug and toxic extrusion (MATE) proteins in the excretion of organic cations, it may be hypothesized that metoclopramide is also a substrate of MATE1/*SLC47A1* and MATE2-K/*SLC47A2*. Both MATE1 and MATE2-K are expressed in the luminal (urine-facing) membrane of kidney proximal tubule cells (25, 28). The genes encoding for OCT1/2 and MATEs are highly polymorphic, which may lead to variability in the pharmacokinetics and clinical response to their substrate drugs (29).

Positron emission tomography (PET) allows monitoring of the tissue concentrations of radiolabeled drugs and has been used to assess the impact of transporters on drug distribution and excretion (30). [^11^C]Metoclopramide has been developed as a PET radiotracer to study P-gp activity at the blood-brain barrier (21–23). The aim of this study was to assess by means of PET imaging with [^11^C]metoclopramide in mice the role of cation transporters (OCTs, MATEs) as possible factors accounting for variability in metoclopramide disposition. To this end, we assessed the dose dependency of [^11^C]metoclopramide disposition and the effect of administration of prototypical cation transporter inhibitors. In addition, to further assess the role of OCTs in the disposition of [^11^C]metoclopramide, *Slc22a1/2*^*(−/−)*^ mice were investigated.

## Materials and Methods

### Chemicals

Unless otherwise stated, all chemicals were purchased from Sigma-Aldrich (Schnelldorf, Germany) or Merck (Darmstadt, Germany). I.v. injection solutions of metoclopramide (Paspertin®, 10 mg/2 mL, Mylan Österreich GmbH, Vienna, Austria), cimetidine (200 mg/2 mL, ratiopharm GmbH, Ulm, Germany) and sulpiride (Dogmatil®, 100 mg/2 mL, Sanofi-Aventis Deutschland GmbH, Frankfurt am Main, Germany GmbH) were obtained from a local pharmacy. For i.v. injection, cimetidine solution was diluted 1:1 with physiological saline solution (0.9%, *w*/*v*) and injected at a volume of 6 μL per g body weight. Sulpiride solution was diluted 1:8 with physiological saline solution and injected at a volume of 4 μL per g body weight.

### Radiotracer Synthesis

[^11^C]Metoclopramide was synthesized as previously described (17). For i.v. injection into mice, [^11^C]metoclopramide was formulated in physiological saline solution. For experiments in which a pharmacological dose of unlabeled metoclopramide was co-injected with [^11^C]metoclopramide, undiluted metoclopramide solution (Paspertin®) was directly added to the formulated radiotracer solution.

### Animals

Female wild-type C57BL/6 mice were obtained from Charles River Laboratories (Sulzfeld, Germany), male and female FVB wild-type mice were obtained from Envigo (Venray, The Netherlands), and male and female *Slc22a1/2*^*(−/−)*^ mice (in an FVB genetic background) were obtained from the central animal facility at the University Hospital Münster (Münster, Germany). At the time of experiment, wild-type animals (C57BL/6 and FVB) were 6–12 weeks old and weighed 20.5 ± 2.9 g and *Slc22a1/2*^*(−/−)*^ mice were 21 weeks old and weighed 28.4 ± 2.6 g. In total, 70 mice were used in the experiments. All animals were housed in type III IVC cages under controlled environmental conditions (21.8 ± 1.0°C, 40% to 70% humidity, 12-h light/dark cycle) with free access to standard laboratory rodent diet (LASQCdiet™, LASvendi, Soest, Germany) and water. An acclimatization period of at least 1 week was allowed before the animals were used in the experiments. The study was approved by the national authorities (Amt der Niederösterreichischen Landesregierung, approval numbers: LF1-TVG-48/043–2019 and LF1-TVG-62/003–2020) and study procedures were in accordance with the European Communities Council Directive of September 22, 2010 (2010/63/EU). The animal experimental data reported in this study are in compliance with the ARRIVE (Animal Research: Reporting in Vivo Experiments) guidelines.

### Experimental Design

Table I summarizes the different groups of animals examined in this study. Five groups of female C57BL/6 wild-type mice underwent 90-min PET scans after i.v. administration of a microdose (< 1 μg) of [^11^C]metoclopramide. Two of these groups received a co-injection with pharmacological doses of unlabeled metoclopramide (5 and 10 mg/kg respectively). The doses of co-injected unlabeled metoclopramide were selected based on previous work by Caillé et al. (17). Two further groups received an i.v. pretreatment with either cimetidine (150 mg/kg) or sulpiride (25 mg/kg) at 5 min before the start of the PET scan. The doses of cimetidine and sulpiride were selected based on previous work by Jensen et al. and Takano et al. (31, 32). Four additional groups of female C57BL/6 mice were used to assess radiolabeled metabolites at 15 min after i.v. injection of [^11^C]metoclopramide without and with co-administration of either unlabeled metoclopramide (5 mg/kg), cimetidine (150 mg/kg), or sulpiride (25 mg/kg). Two groups of male and female FVB wild-type (*n* = 4/4) and *Slc22a1/2*^*(−/−)*^ mice (*n* = 3/3) underwent 15-min PET scans after i.v. administration of [^11^C]metoclopramide followed by blood sampling and organ collection to assess radiolabeled metabolites of [^11^C]metoclopramide.

**Table I:** Overview of Animal Groups Examined in the Study

Study type	Genetic background; sex	Group	*n*
PET ^a^	C57BL/6; female	Baseline	5
		Metoclopramide (5 mg/kg)	6
		Metoclopramide (10 mg/kg)	4
		Cimetidine (150 mg/kg)	4
		Sulpiride (25 mg/kg)	6
Metabolism ^b^	C57BL/6; female	Control	4
		Metoclopramide (5 mg/kg)	6
		Cimetidine (150 mg/kg)	4
		Sulpiride (25 mg/kg)	4
PET + metabolism ^c^	FVB; male/female	Wild-type	4/4
		*Slc22a1/2*^*(−/−)*^	3/3

^a^PET scan lasted 90 min

^b^Metabolites in the organs and fluids were measured at 15 min after radiotracer injection. Animals did not undergo a PET scan

^c^PET scan lasted 15 min, then organs and fluids from all scanned animals were collected and metabolites were measured

### Metabolism

Different animal groups (Table I) were i.v. injected under isoflurane/air anesthesia with [^11^C]metoclopramide (28 ± 12 MBq, corresponding to 0.4 ± 0.4 μg of unlabeled metoclopramide). At 15 min after radiotracer injection, blood was collected from the retro-bulbar plexus and animals were killed by cervical dislocation while under deep anesthesia. Blood was centrifuged to obtain plasma, the liver and the kidneys were removed and urine was collected. Proteins were precipitated by the addition of acetonitrile (1 μL/μL plasma; 1000 μL for the liver, 200 μL for the kidneys and 0.5 μL/μL urine). All solutions were vortexed and centrifuged. Each supernatant (plasma, liver, kidneys and urine, 5 μL each) and diluted [^11^C]metoclopramide solution as a reference were spotted on thin-layer chromatography (TLC) plates (silica gel 60F 254 nm, 10 × 20 cm; Merck, Darmstadt, Germany) and the plates were developed in ethyl acetate/ethanol/ammonium hydroxide (25%, *w*/*v*) (80/20/5, *v*/*v*/*v*). Detection was performed by placing the TLC plates on multisensitive phosphor screens (PerkinElmer Life Sciences, Waltham, MA). The screens were scanned at 300 dpi resolution using a PerkinElmer Cyclone® Plus Phosphor Imager (Perkin-Elmer Life Sciences). The retardation factor (R_f_) for [^11^C]metoclopramide was 0.6, while the radiolabeled metabolites remained on the start (R_f_ = 0).

### PET Imaging

PET imaging was performed under isoflurane/air anesthesia and animals were warmed throughout the experiment while constantly monitoring body temperature and respiratory rate. A microPET Focus220 scanner (Siemens Medical Solutions, Knoxville, TN, USA) was used for PET imaging. [^11^C]Metoclopramide was administered in a volume of 100 μL as an i.v. bolus (34 ± 8 MBq, corresponding to 0.2 ± 0.1 μg of unlabeled metoclopramide). A 90-min or 15-min dynamic PET scan was initiated at the start of radiotracer injection for the C57BL/6 mouse groups and for the FVB mouse groups, respectively. List mode data were acquired with a timing window of 6 ns and an energy window of 250–750 keV. At the end of the PET scans, blood was collected from the retro-bulbar plexus and animals were killed by cervical dislocation. Blood was centrifuged to obtain plasma and aliquots of blood and plasma were counted for radioactivity in a gamma counter. In the FVB mouse groups, organs and fluids were collected at the end of the 15-min PET scan and analyzed for radiolabeled metabolites of [^11^C]metoclopramide as described above.

### PET Data Analysis

The PET data from the C57BL/6 mice were sorted into 25 time frames, the duration of which increased from 5 s to 20 min, and the PET data from the FVB mice were sorted into 18 time frames with a duration increasing from 5 s to 5 min. PET images were reconstructed using Fourier re-binning of the 3-dimensional sinograms followed by a 2-dimensional filtered back-projection with a ramp filter giving a voxel size of 0.4 × 0.4 × 0.796 mm^3^. Using the medical image data examiner software AMIDE (33), the left ventricle of the heart (image-derived arterial blood curve), brain, liver, left kidney and urinary bladder were manually outlined as regions of interest on the PET images, guided by representative magnetic resonance images obtained for a few animals on a 1-T benchtop MR scanner (ICON; Bruker BioSpin GmbH), to derive concentration-time curves expressed in units of percent injected dose per mL (%ID/mL).

### Kinetic Analysis

The area under the concentration-time curves (AUC, %ID/mL x min) was calculated for each region of interest using Prism 8 Software (GraphPad, La Jolla, CA, USA). The kidney and liver uptake rate constants (*k*_uptake,kidney_ and *k*_uptake,liver_, respectively - mL/min/mL tissue) of radioactivity were estimated from 0.4 to 4 min after [^11^C]metoclopramide injection by integration plot analysis (34):

$$ \frac{{\mathrm{X}}_{\mathrm{t},\mathrm{tissue}}}{{\mathrm{C}}_{\mathrm{t},\mathrm{blood}}}={k}_{\mathrm{uptake},\mathrm{tissue}}\ \mathrm{x}\ \frac{{\mathrm{AUC}}_{0-\mathrm{t},\mathrm{blood}}}{{\mathrm{C}}_{\mathrm{t},\mathrm{blood}}}+{\mathrm{V}}_{\mathrm{E},\mathrm{tissue}} $$

where X_t,tissue_ is the amount of radioactivity per mL tissue in the kidney or liver at time t, C_t,blood_ is the radioactivity concentration in the blood (image-derived curve from the region of interest placed in the left ventricle of the heart) at time t, and AUC_0-t,blood_ is the area under the concentration-time curve of the left ventricle of the heart from time 0 to time t. V_E_ corresponds to the y-intercept of the integration plot. *k*_uptake,tissue_ is obtained by performing linear regression analysis of a plot of X_t,tissue_/C_t,blood_ versus AUC_0-t,blood_/C_t,blood_ and calculating the slope of the regression line. The intrinsic urinary excretion clearance of total radioactivity (CL_int,urine_, mL/min) was calculated for the animals which underwent 90-min PET scans from 30 to 70 min after radiotracer injection using integration plot analysis (34) as follows:

$$ {\mathrm{X}}_{\mathrm{t},\mathrm{urine}}={\mathrm{CL}}_{\operatorname{int},\mathrm{urine}}\ \mathrm{x}\ {\mathrm{AUC}}_{0-\mathrm{t},\mathrm{kidney}}+{\mathrm{V}}_{\mathrm{E}} $$

where X_t,urine_ is the total amount of radioactivity excreted in urine at time t, and AUC_0-t,kidney_ is the area under the concentration-time curve of the kidney from time 0 to time t. V_E_ is the y-intercept of the integration plot. CL_int,urine_ was obtained by performing linear regression analysis of a plot of X_int,urine_ versus AUC_0-t,kidney_ and calculating the slope of the regression line.

The total radioactivity concentration ratios (kidney/plasma, liver/plasma and urine/kidney) were calculated using the following equation:

$$ \mathrm{Concentration}\ \mathrm{ratio}=\frac{{\mathrm{Concentration}}_{\mathrm{total}\ \mathrm{PET},\kern0.5em \mathrm{tissue}1}}{{\mathrm{Concentration}}_{\mathrm{total}\ \mathrm{PET},\kern0.5em \mathrm{tissue}2}} $$

where Concentration_total PET,tissue1_ and Concentration_total PET,tissue2_ are the %ID/mL values of total radioactivity obtained at 15 min after radiotracer injection from the PET images. The plasma concentration was obtained by multiplying the concentration in the left ventricle of the heart by the plasma/blood radioactivity ratio, determined from the blood sample collected at the end of the PET scan.

Since [^11^C]metoclopramide is highly metabolized in mice, total radioactivity was corrected for radiolabeled metabolites in order to calculate the concentration ratios of unchanged [^11^C]metoclopramide. The correction for metabolites of the total radioactivity concentration in each organ was performed using the following equation:

$$ {\mathrm{Concentration}}_{\mathrm{metabolite}-\mathrm{corrected}}={\mathrm{Concentration}}_{\mathrm{total}\ \mathrm{PET}}\ \mathrm{x}\ {\mathrm{P}}_{\mathrm{unchanged}}/100 $$

where Concentration_total PET_ is the %ID/mL value of total radioactivity obtained at 15 min after radiotracer injection from the PET images and P_unchanged_ is the average percentage of unchanged [^11^C]metoclopramide in each sampled organ or fluid (Table II). No metabolite data at 15 min after radiotracer injection were available for the PET scanned animals with C57BL/6 genetic background, since the PET scan lasted 90 min; thus, P_unchanged_ was used from the separate cohort of C57BL/6 animals which did not undergo a PET scan (Table I). For the PET scanned animals with FVB genetic background, P_unchanged_ was measured at the end of the 15-min PET scans for all animals.

**Table II:** Percentage of Unchanged [^11^C]Metoclopramide in Different Organs or Fluids Determined with Radio-TLC Analysis at 15 Min After Radiotracer Injection

Group		*n*	Organ			
Genetic back-ground; sex			Plasma	Kidney	Urine	Liver
C57BL/6; female	Baseline	4	18 ± 2	64 ± 6	29 ± 4	57 ± 4
	Metoclopramide (5 mg/kg)	6	37 ± 4	74 ± 4	43 ± 7	76 ± 4
	Cimetidine (150 mg/kg)	4	89 ± 2***	96 ± 2**	87 ± 4**	96 ± 2***
	Sulpiride (25 mg/kg)	4	37 ± 11	75 ± 5	36 ± 5	76 ± 7
FVB; male/female	Wild-type	4/4	23 ± 7	70 ± 20	64 ± 22	69 ± 6
	*Slc22a1/2*^*(−/−)*^	3/3	25 ± 6	72 ± 8	48 ± 10	74 ± 9

Percentage of unchanged radiotracer is given as mean ± SD. ** *p* < 0.01, *** *p* < 0.001, Kruskal-Wallis followed by a Dunn’s multiple comparison test against a reference group (baseline)

### Statistical Analysis

Statistical analysis was done using Prism 8 software. Differences between two groups were analyzed by a Mann-Whitney test, and differences between multiple groups were analyzed by Kruskal-Wallis tests followed by a Dunn’s multiple comparison test against the baseline group. The level of statistical significance was set to *p* ≤ 0.05.

## Results

**Fig. 1:**
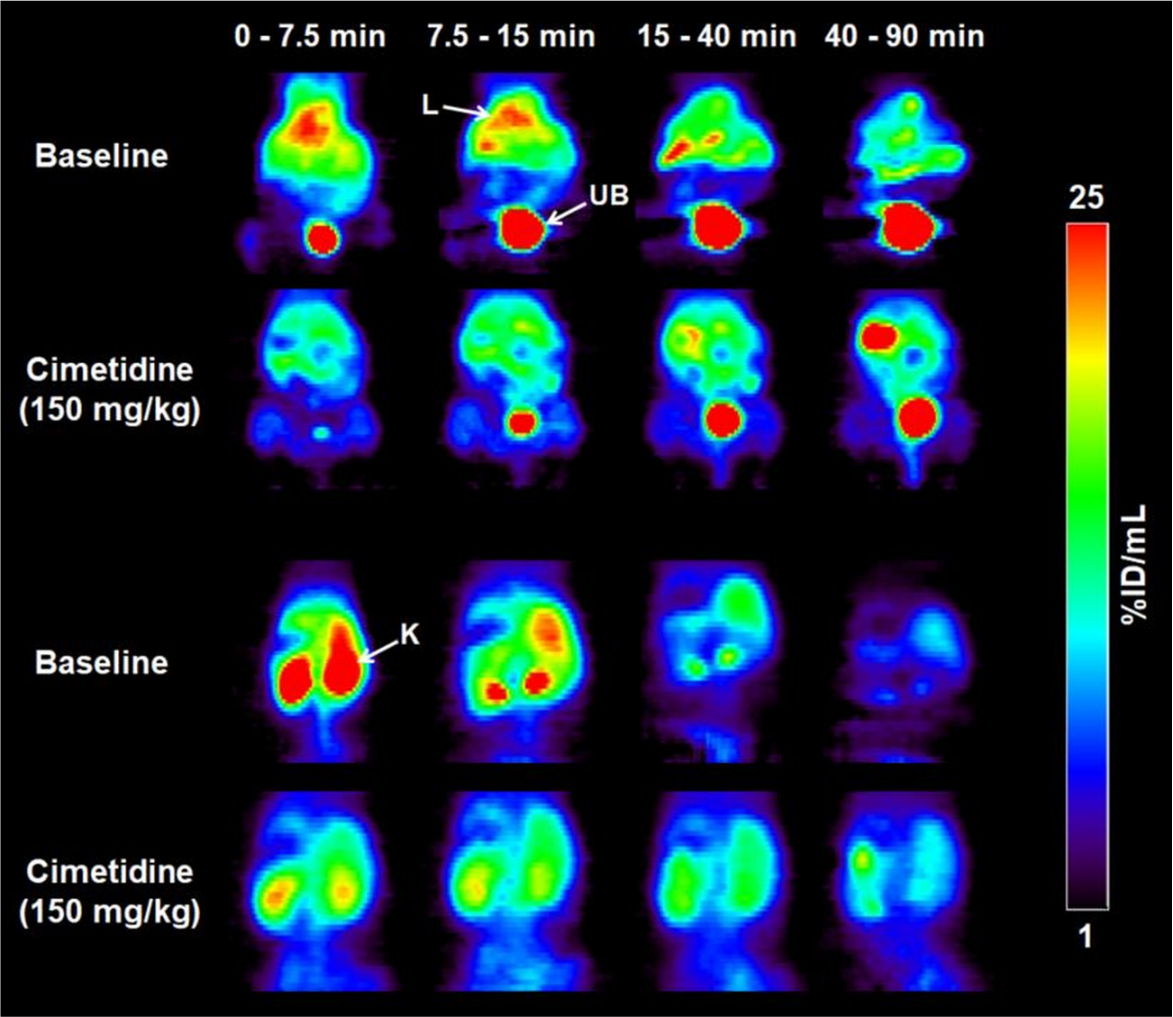
Serial PET images of a representative C57BL/6 wild-type mouse without (baseline) and with pretreatment with cimetidine (150 mg/kg) at 5 min before injection of [^11^C]metoclopramide. Two different coronal views are shown in order to visualize all the excretory organs. Radioactivity concentration is expressed as percent of the injected dose per mL (%ID/mL). Anatomical structures are labeled with white arrows (K: kidney; L: liver; UB: urinary bladder).

**Fig. 2:**
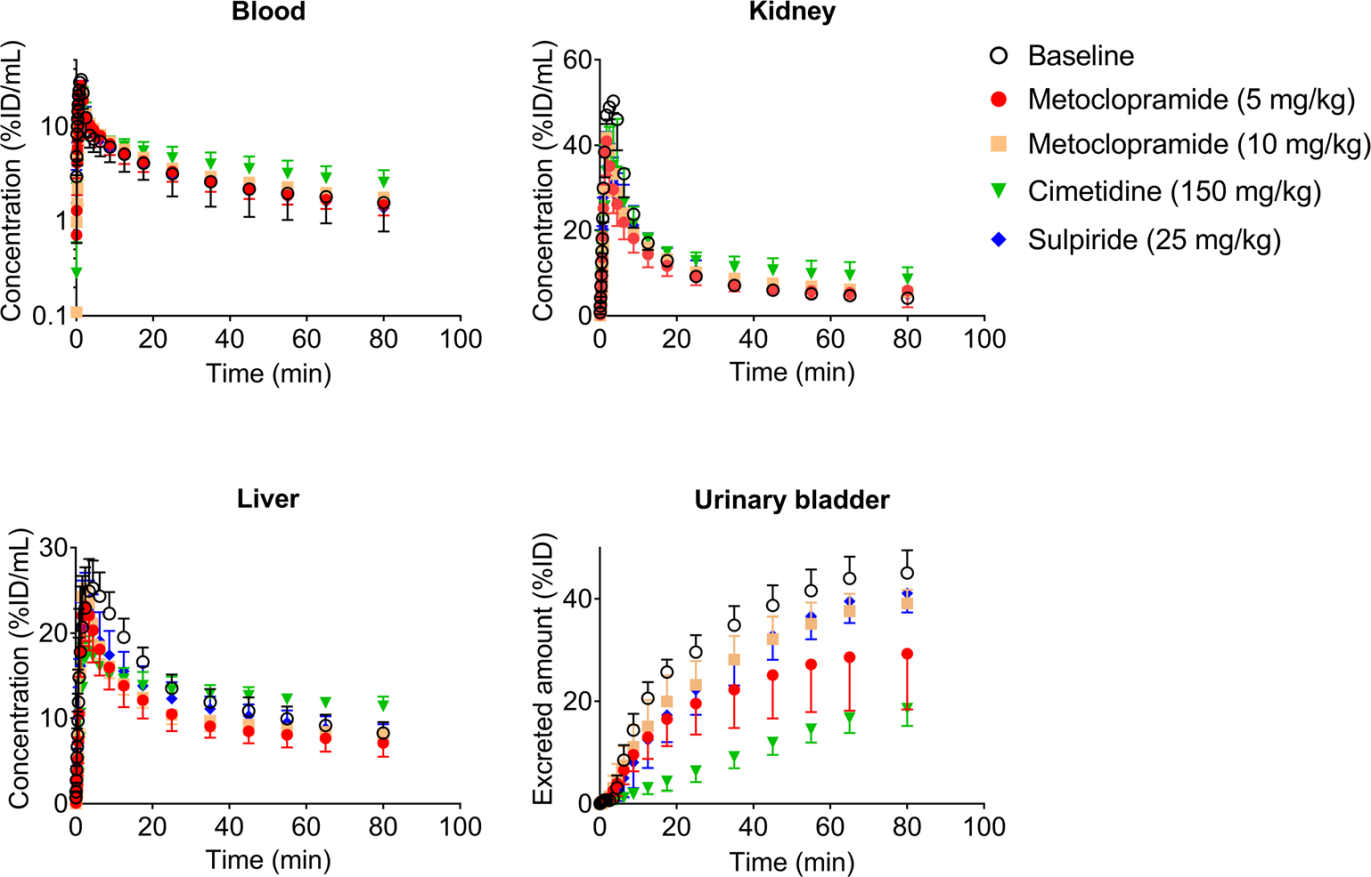
Mean time-radioactivity curves (%ID/mL or %ID ± SD) in the blood (image-derived blood curve from the left ventricle of the heart), left kidney, liver and urinary bladder of wild-type C57BL/6 mice for the baseline group, and groups treated with 5 mg/kg or 10 mg/kg metoclopramide, 150 mg/kg cimetidine or 25 mg/kg sulpiride.

In this study we used PET to study the influence of cation transporters on the renal and hepatobiliary disposition of [^11^C]metoclopramide. First, the dose dependency of [^11^C]metoclopramide disposition and the effect of prototypical cation transporter inhibitors (cimetidine and sulpiride) were investigated. We further assessed metabolism of [^11^C]metoclopramide by analyzing extracts from different organs and fluids collected at 15 min after [^11^C]metoclopramide injection, either in separate groups of mice or in the same animals that underwent PET imaging (see Table I) with radio-TLC. These experiments revealed that [^11^C]metoclopramide was metabolized to a significant extent at 15 min after i.v. administration (Table II). Cimetidine significantly increased the percentage of unchanged [^11^C]metoclopramide in all analyzed organs and fluids, while unlabeled metoclopramide and sulpiride also showed a trend towards increasing the percentage of unchanged [^11^C]metoclopramide, but without reaching statistical significance (Table II). Serial PET images of a representative C57BL/6 wild-type mouse (Fig. 1) showed that after i.v. injection of [^11^C]metoclopramide, radioactivity was rapidly taken up by the kidneys and the liver. After some time, radioactivity was excreted into the urinary bladder, while the intestine was hardly identifiable on the PET images. Time-radioactivity curves for the selected organs are shown in Fig. 2. Maximum concentration of radioactivity in the kidneys and liver was reached within 5 min after [^11^C]metoclopramide injection and corresponded to amounts of approximately 17% and 36% of the injected dose, respectively, assuming average mouse organ volumes published in the literature (35). The amount of radioactivity excreted into the urinary bladder at the end of the 90-min PET scan corresponded to approximately 45% of the injected dose. As compared to the other studied organs, differences in the PET-derived urinary bladder kinetics between groups appeared to be more pronounced, especially after pretreatment with cimetidine as compared to the baseline group (Figs. 1 and 2).

**Fig. 3:**
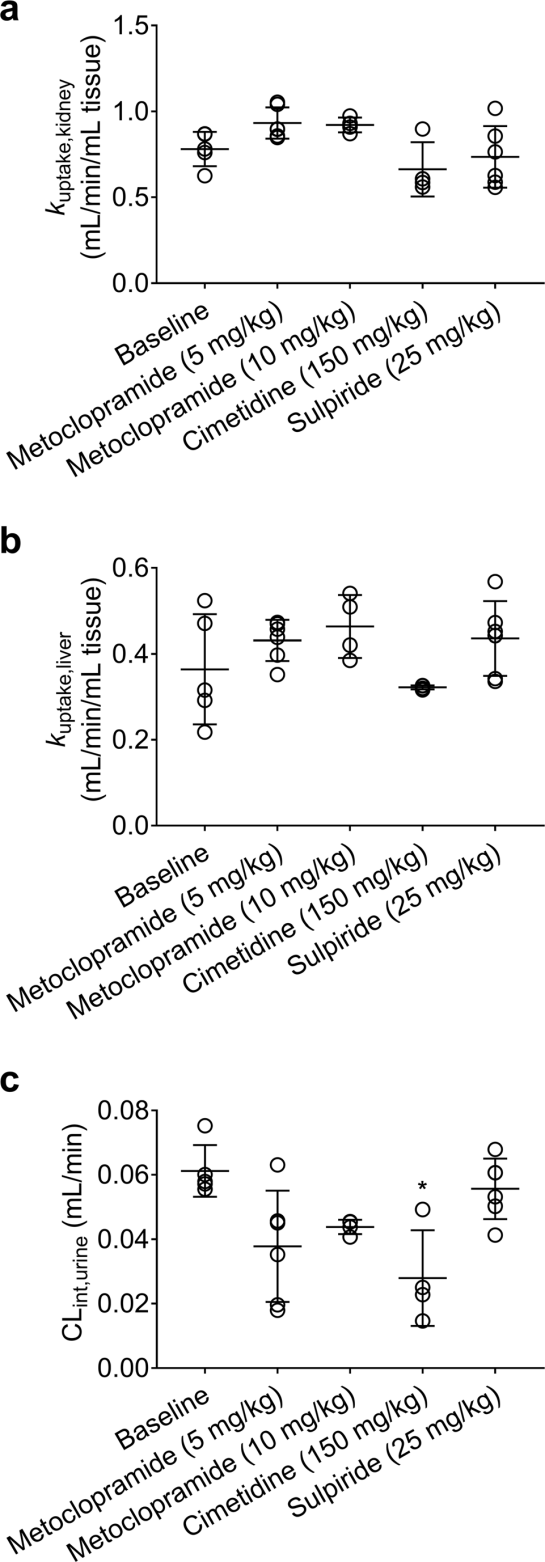
Kidney (**a**) and liver (**b**) uptake rate constants and intrinsic urinary excretion clearance (**c**) of total radioactivity in C57BL/6 wild-type mice (baseline), wild-type mice co-injected with unlabeled metoclopramide (5 and 10 mg/kg) and wild-type mice pretreated with cimetidine (150 mg/kg) or sulpiride (25 mg/kg). * *p* < 0.05, Kruskal-Wallis followed by Dunn’s multiple comparison test against the reference (baseline) group.

Integration plot analysis was used to estimate the rate constants for uptake of total radioactivity from blood into the kidneys (*k*_uptake,kidney_) or into the liver (*k*_uptake,liver_). *k*_uptake,kidney_ values were in the range of 0.5–1 mL/min/mL kidney (Fig. 3a), which was below the kidney blood flow rate in mice (3.8 mL/min/mL kidney) (35). *k*_uptake,liver_ values ranged from 0.2–0.6 mL/min/mL liver (Fig. 3b), which was lower than the liver blood flow rate in mice (1.4 mL/min/mL liver) (35). No significant differences in *k*_uptake,kidney_ (*p* = 0.3873 and 0.2554 for 5 and 10 mg/kg metoclopramide co-administration, respectively and *p* > 0.9999 for cimetidine and sulpiride pretreatment) and *k*_uptake,liver_ (*p* = 0.7931, 0.4383 and 0.7422 for 5 and 10 mg/kg metoclopramide and sulpiride co-administration, respectively, and *p* > 0.9999 for cimetidine pretreatment) were observed between the different treatment groups and the baseline group (Fig. 3a and b).

**Fig. 4:**
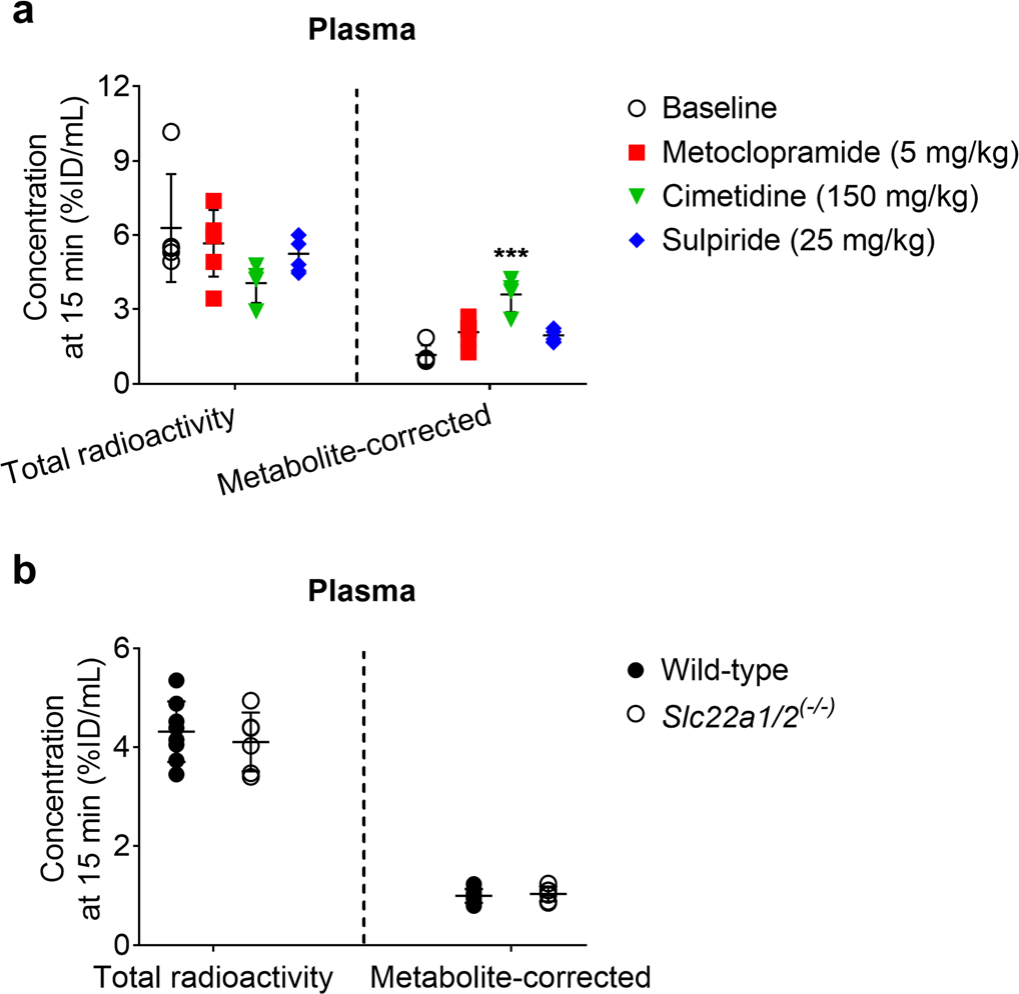
Total (left side of each panel) and metabolite-corrected radioactivity concentrations (right side of each panel, corresponding to unchanged [^11^C]metoclopramide) in the plasma of C57BL/6 at 15 min after [^11^C]metoclopramide injection in wild-type mice without and with co-administration of metoclopramide (5 or 10 mg/kg) or pretreatment with cimetidine (150 mg/kg) or sulpiride (25 mg/kg) (**a**) and in wild-type and *Slc22a1/2*^*(−/−)*^ FVB mice (**b**). *** *p* < 0.001, Kruskal-Wallis followed by a Dunn’s multiple comparison test against the reference (baseline) group.

**Fig. 5:**
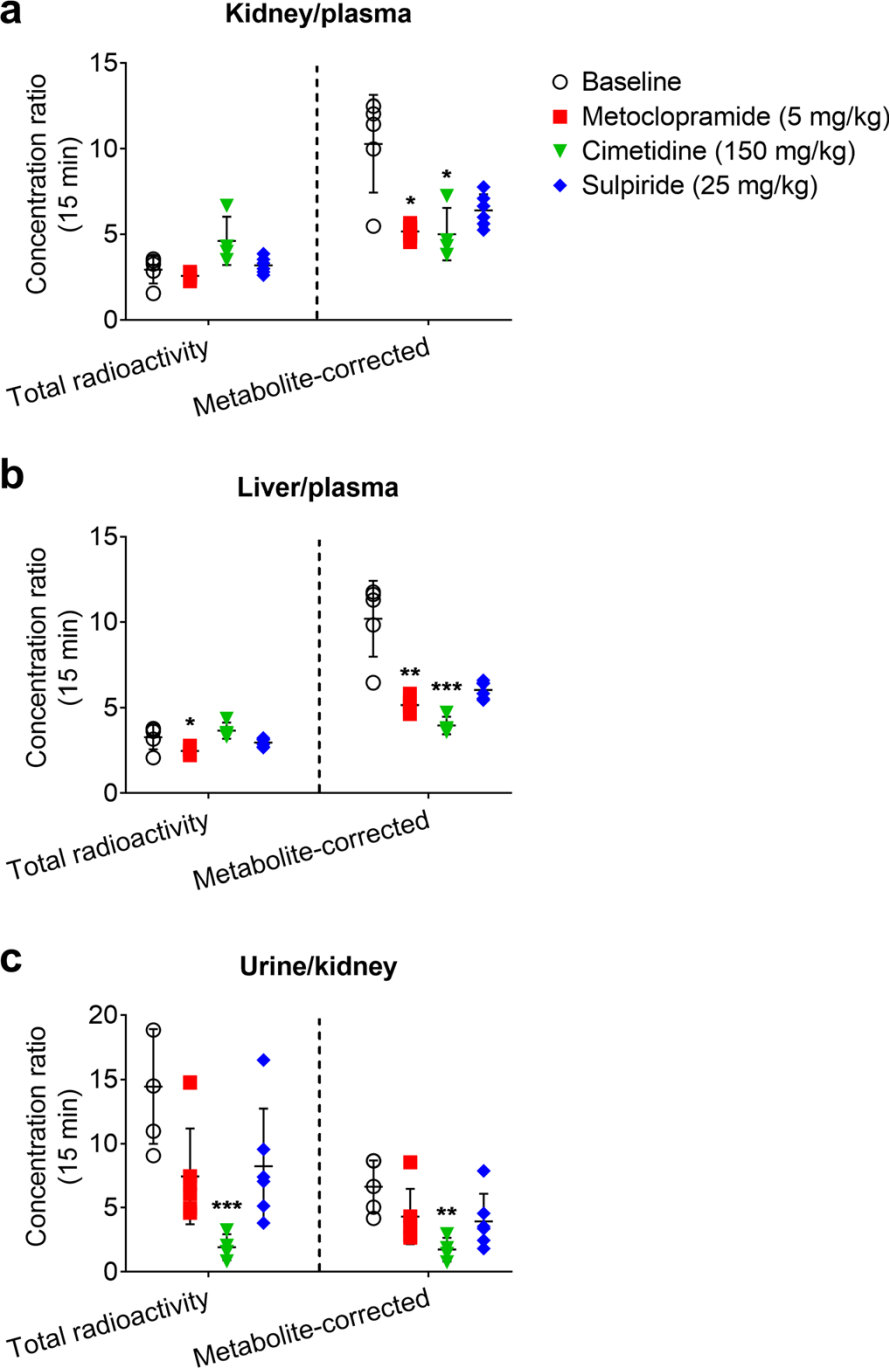
Kidney/plasma (**a**), liver/plasma (**b**) and urine/kidney (**c**) concentration ratios calculated at 15 min after radiotracer injection. Differences between groups were assessed from total PET radioactivity (left side of each panel) or from PET radioactivity corrected for radiolabeled metabolites (right side of each panel, corresponding to unchanged [^11^C]metoclopramide). * *p* < 0.05, ** *p* < 0.01, *** *p* < 0.001, Kruskal-Wallis followed by a Dunn’s multiple comparison test against the reference (baseline) group.

Due to the differences in [^11^C]metoclopramide metabolism between groups, it was not straightforward to distinguish transporter effects from metabolism effects from the PET data alone. Therefore, PET-derived total radioactivity concentrations in different organs and fluids at the time point of 15 min after radiotracer injection were corrected for radiolabeled metabolites of [^11^C]metoclopramide determined with radio-TLC analysis (Table II). This correction revealed up to 3-fold increases in the concentration of unchanged [^11^C]metoclopramide in plasma for all investigated treatments as compared with the baseline group, which was statistically significant in the case of cimetidine (*p* = 0.0007) (Fig. 4a). We next determined concentration ratios of unchanged [^11^C]metoclopramide (kidney/plasma, liver/plasma, and urine/kidney) for the 15 min time point (Fig. 5). Co-administration with 5 mg/kg metoclopramide and with cimetidine caused a significant decrease in the uptake of unchanged [^11^C]metoclopramide from plasma into both kidney (Fig. 5a) (*p* = 0.0165 and 0.0132, respectively) and liver (Fig. 5b) (*p* = 0.0091 and 0.0003, respectively).

Cimetidine pretreatment caused a significant reduction (*p* = 0.0207) in the intrinsic urinary excretion clearance of total radioactivity (CL_int,urine_) (Fig. 3c). There was also a trend towards a decrease in CL_int,urine_ after co-administration of 5 mg/kg and 10 mg/kg of metoclopramide, but statistical significance was not reached. In addition, after pretreatment with cimetidine there was a reduction in the urine/kidney concentration ratio at 15 min after radiotracer injection, which was more pronounced for total (*p* = 0.0009) than for metabolite-corrected radioactivity concentrations (*p* = 0.0031) (Fig. 5c).

**Fig. 6:**
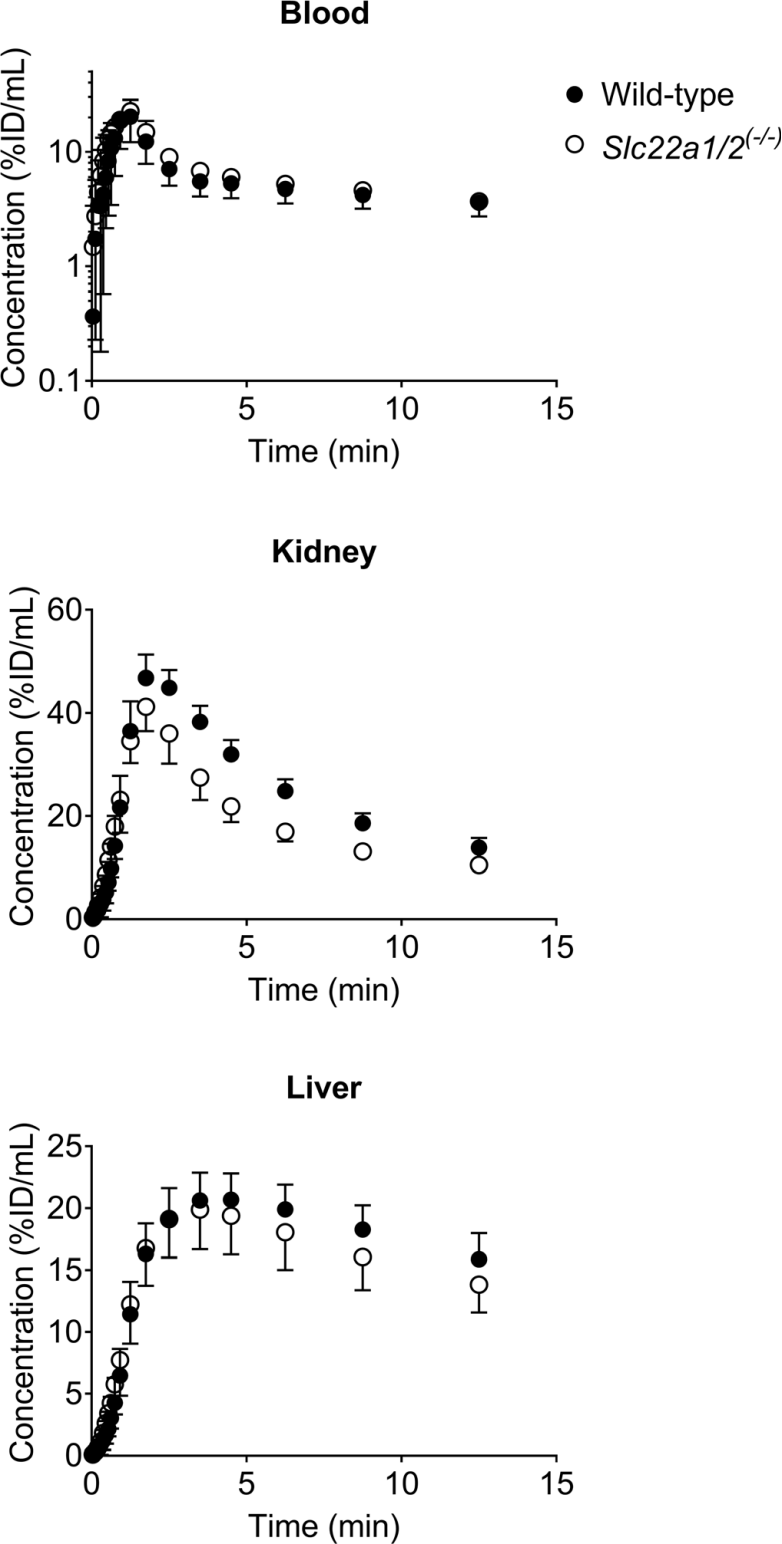
Mean concentration-time curves (%ID/mL ± SD) in the blood (image-derived blood curve), left kidney and liver of FVB wild-type and *Slc22a1/2*^*(−/−)*^ mice.

**Fig. 7:**
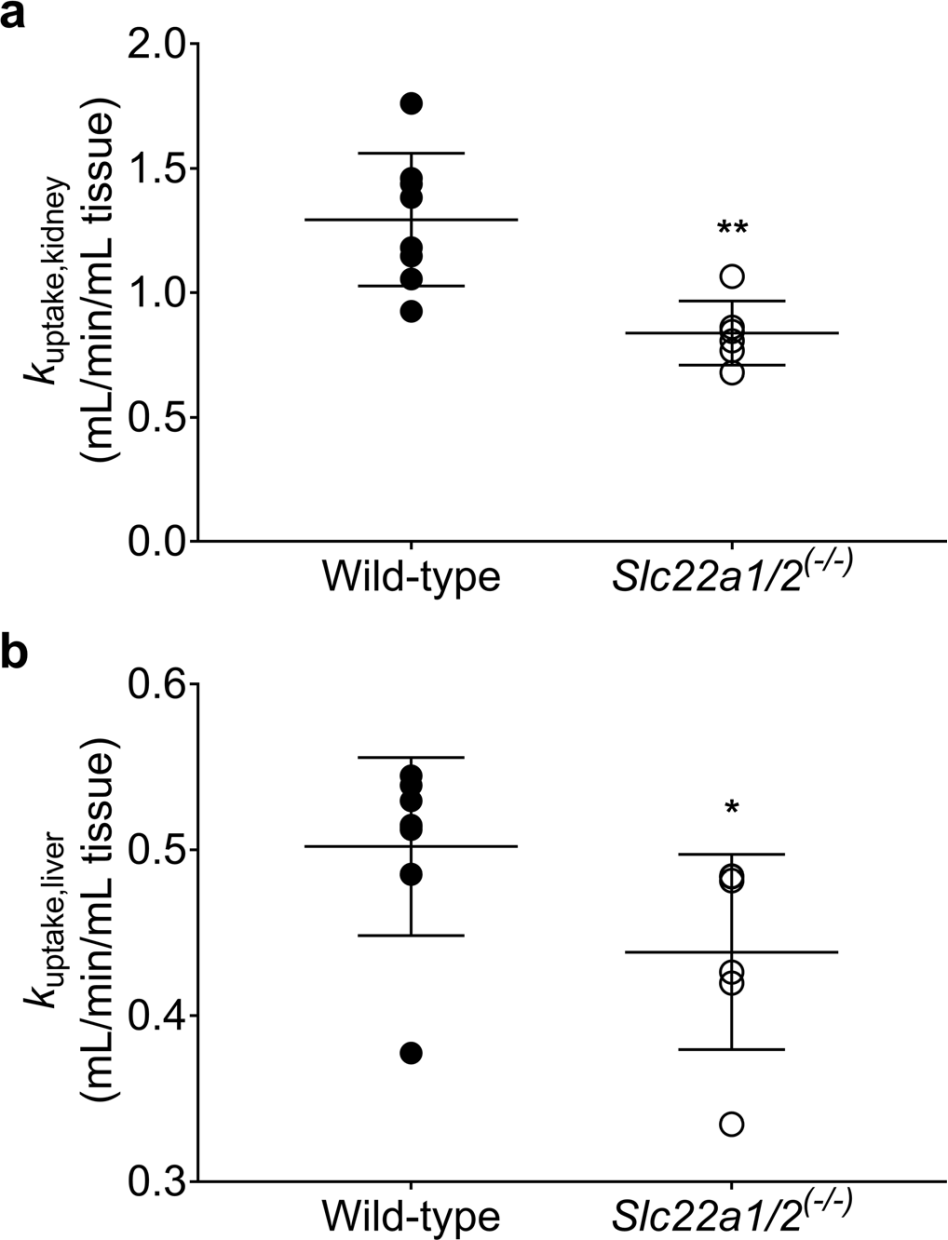
Kidney (**a**) and liver (**b**) uptake rate constants of total radioactivity in FVB wild-type and *Slc22a1/2*^*(−/−)*^ mice. * *p* < 0.05, ** *p* < 0.01, Mann-Whitney test.

**Fig. 8:**
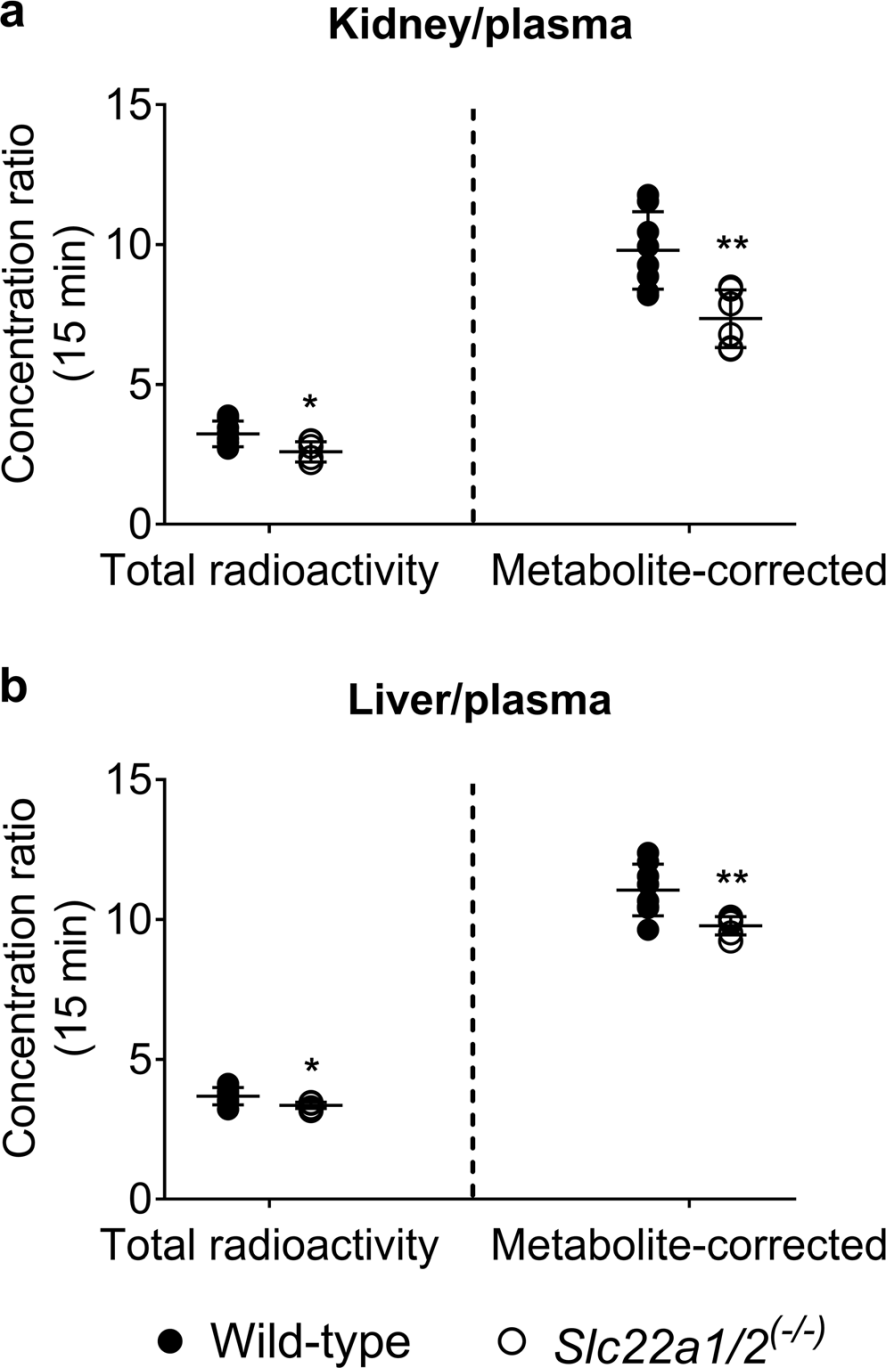
Kidney/plasma (**a**) and liver/plasma (**b**) concentration ratios calculated at 15 min after radiotracer injection. Differences between groups were assessed from total PET radioactivity (left side of each panel) or from PET radioactivity corrected for radiolabeled metabolites (right side of each panel, corresponding to unchanged [^11^C]metoclopramide). * *p* < 0.05, ** *p* < 0.01, Mann-Whitney test.

The correction of the PET data at 15 min for radiolabeled metabolites revealed that all treatments decreased the uptake of unchanged [^11^C]metoclopramide from plasma into both kidney and liver (Fig. 5a and b), which suggested an involvement of mouse OCT1 and OCT2. Thus, in order to further investigate the role of these uptake transporters in the kidney and liver uptake of [^11^C]metoclopramide, we performed 15-min PET scans with [^11^C]metoclopramide in FVB wild-type and *Slc22a1/2*^*(−/−)*^ mice. [^11^C]Metoclopramide was metabolized to a similar extent in both mouse strains (Table II). Concentration-time curves for the different organs of wild-type and *Slc22a1/2*^*(−/−)*^ mice are shown in Fig. 6. The concentration of unchanged [^11^C]metoclopramide in plasma at 15 min after injection was not significantly different between the two mouse genotypes (Fig. 4b) (*p* = 0.5941 and 0.6417 for total radioactivity and metabolite-corrected data, respectively). *k*_uptake,kidney_ and *k*_uptake,liver_ of total radioactivity were significantly lower (*p* = 0.0027 and 0.0127, respectively) in *Slc22a1/2*^*(−/−)*^ mice than in wild-type mice (Fig. 7). Both kidney/plasma and liver/plasma concentration ratios calculated at 15 min after radiotracer injection were significantly lower in the knockout animals (kidney/plasma: *p* = 0.0246 and 0.0080; liver/plasma: *p* = 0.0127 and 0.0080 for total radioactivity and metabolite-corrected data, respectively), independent of metabolite correction (Fig. 8).

**Fig. 9:**
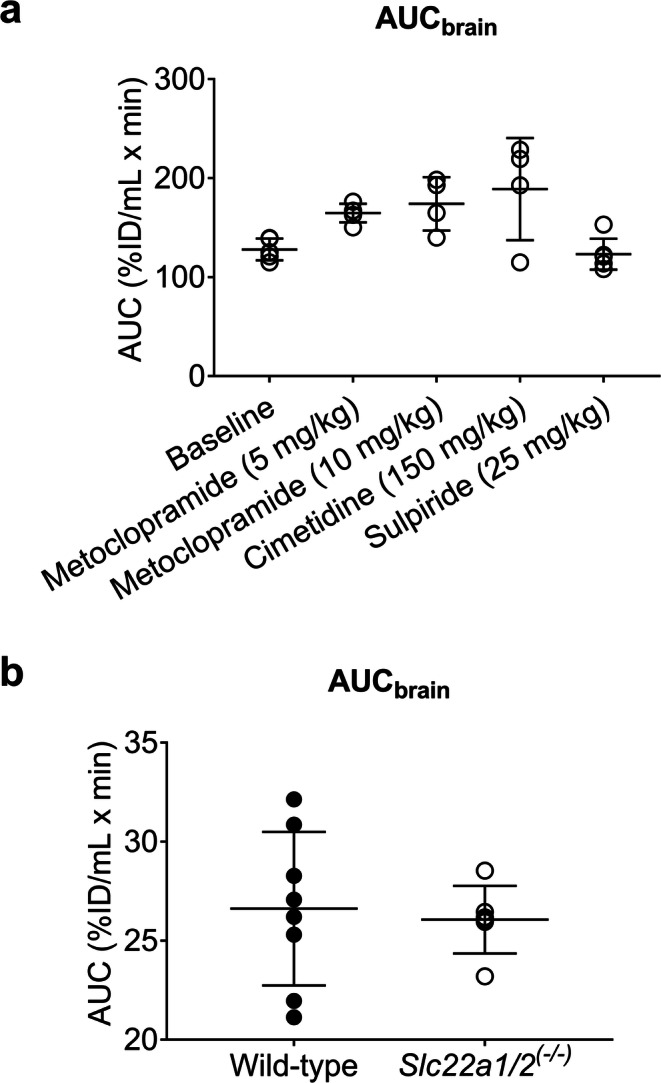
(**a**) Area under the brain concentration-time curve of total radioactivity (AUC_brain_, %ID/mL x min) in wild-type C57BL/6 mice without and with co-administration of metoclopramide (5 or 10 mg/kg) or pretreatment with cimetidine (150 mg/kg) or sulpiride (25 mg/kg). (**b**) AUC_brain_ values in wild-type and *Slc22a1/2*^*(−/−)*^ FVB mice.

We also determined AUC_brain_ values in all examined groups as a measure of brain exposure to [^11^C]metoclopramide (Fig. 9). AUC_brain_ showed a trend towards an increase for the 5 mg/kg and 10 mg/kg metoclopramide and for the cimetidine pretreated groups as compared to baseline, but statistical significance was not reached (*p* = 0.0802, 0.0514 and 0.0912, respectively). This trend was not observed for the sulpiride pretreated group (*p* > 0.9999). AUC_brain_ did not differ between *Slc22a1/2*^*(−/−)*^ and wild-type mice (Fig. 9b).

## Discussion

In the present study we used PET imaging in mice to assess the involvement of organic cation transporters (OCTs, MATEs) in the disposition of the radiolabeled antiemetic drug [^11^C]metoclopramide. This is of relevance as metoclopramide shows considerable inter-individual variability in its efficacy and side effects, and previous studies have provided evidence for non-linear, dose-dependent pharmacokinetics of metoclopramide in rats and humans (9, 14–16). Non-linear pharmacokinetics of metoclopramide were observed both after oral and i.v. administration, whereby effects appeared to be more pronounced after oral administration. Drug transporters are widely accepted determinants of drug disposition (25), and previous observations related to metoclopramide pharmacokinetics and pharmacodynamics may therefore be related to the effects of drug transporters.

Our data revealed that after i.v. injection into mice, [^11^C]metoclopramide undergoes metabolism in the liver to a significant extent followed by excretion of its radiolabeled metabolites into the urine, and that only a smaller portion of the drug is excreted in unchanged form into the urine. With a predominant clearance by metabolism, [^11^C]metoclopramide differs from several previously investigated PET tracers for drug transporters, which were hardly metabolized and underwent mainly transporter-mediated excretion of the unchanged parent drug (30). As PET measures total radioactivity in tissue and cannot distinguish different chemical forms of radioactivity, the in vivo behavior of [^11^C]metoclopramide proved very challenging for PET analysis of its clearance pathways. We managed to overcome this limitation by combining the PET experiments with metabolism experiments, which allowed us to correct PET-derived tissue concentrations of total radioactivity at one selected time point of the experiments (i.e. at 15 min after radiotracer injection) for the contribution of radiolabeled metabolites. This approach enabled us to separate transporter effects from metabolism effects. Mice have a very small total blood volume (approximately 1.7 mL), which makes it challenging to perform the repeated blood sampling needed for pharmacokinetic analysis. We therefore generated a PET image-derived blood curve from the left ventricle of the heart. PET-derived radioactivity concentrations in the left ventricle of the heart showed an excellent correlation with blood radioactivity concentrations measured in a gamma counter at the end of the PET scan (*r* = 0.9351, *p* < 0.0001), which validated our approach to generating an image-derived blood curve in mice.

As a first step, we evaluated the dose dependency of [^11^C]metoclopramide pharmacokinetics by comparing [^11^C]metoclopramide disposition after administration of a microdose (< 1 μg) with the co-injection of two different pharmacological doses of unlabeled metoclopramide (5 and 10 mg/kg). No dose dependency could be found for PET-derived concentration-time curves of total radioactivity in blood and different tissues (Fig. 2). However, metabolite correction of the PET data at the time point of 15 min after radiotracer injection revealed that co-administration of a pharmacological dose of metoclopramide (5 mg/kg) significantly decreased kidney/plasma and liver/plasma concentration ratios of unchanged [^11^C]metoclopramide (Fig. 5a,b). The decrease in the liver/plasma concentration ratio of unchanged [^11^C]metoclopramide was consistent with the previously reported decrease in the uptake rate constant of [^11^C]metoclopramide from plasma into the liver (*k*_uptake,liver_) in rats co-injected with 3 mg/kg of metoclopramide, determined using a metabolite-corrected, sampled arterial plasma input function (17). This supported the notion that pharmacological doses of metoclopramide lead to saturation of hepatic and renal uptake transporters.

We then aimed at identifying potential transporters involved in the tissue distribution and excretion of metoclopramide. Metoclopramide has been shown to be an in vitro substrate of human OCT1 and OCT2 (24). Therefore, the effect of two different prototypical cation transporter inhibitors on the disposition of [^11^C]metoclopramide was investigated. The first inhibitor was cimetidine, which was shown to be an inhibitor of mouse MATEs (inhibition constant *K*_i_: 1–4 μM) and mouse OCTs (*K*_i_: 54–143 μM) (36), and which has been successfully used at a dose of 150 mg/kg in a PET study in mice to reveal the contribution of mouse MATEs and OCTs to the liver and kidney disposition of the radiolabeled oral antidiabetic drug [^11^C]metformin (31). After pretreatment with cimetidine, we found a significant decrease in the kidney/plasma and liver/plasma ratios of unchanged [^11^C]metoclopramide at 15 min after injection and an approximately 3-fold increase in the plasma concentration of [^11^C]metoclopramide, which supported an involvement of OCT1 and OCT2 in the kidney and liver uptake of [^11^C]metoclopramide. In addition to its effect on kidney and liver uptake of [^11^C]metoclopramide, cimetidine pretreatment significantly decreased the urinary excretion of [^11^C]metoclopramide and its radiolabeled metabolites (Fig. 3c and 5c), which pointed to an involvement of mouse MATEs. Since [^11^C]metoclopramide is also a substrate of P-gp, this effect may also be attributed to an inhibition of P-gp-mediated urinary excretion of radioactivity by cimetidine. However, although cimetidine is a substrate of P-gp, it has not been classified as a P-gp inhibitor (37). To assess a possible role of P-gp in the urinary excretion of [^11^C]metoclopramide, it would be necessary to examine *Abcb1a/b*^*(−/−)*^ mice. The second investigated cation transporter inhibitor was sulpiride, which has been shown to significantly inhibit OCT1-mediated liver uptake of a PET microdose of [^11^C]sulpiride in humans at a single oral dose of approximately 8 mg/kg (32). Pretreatment of mice with sulpiride (25 mg/kg) tended to decrease the kidney/plasma and liver/plasma ratios of unchanged [^11^C]metoclopramide, but without reaching statistical significance. No effect of sulpiride on urinary excretion of radioactivity was observed.

We further observed that cimetidine dramatically increased the percentage of unchanged [^11^C]metoclopramide in each analyzed tissue and fluid (Table II). This suggests a reduction in the metabolism of [^11^C]metoclopramide, which can be most likely explained by the inhibition of metabolizing enzymes. Metoclopramide is mainly metabolized by CYP2D6, and to a lesser extent by CYP3A (10, 11). It has been estimated that 44% of total clearance of orally administered metoclopramide is accounted for by CYP2D6 metabolism in humans (38). Cimetidine is a potent CYP2D6 and CYP3A inhibitor (39), suggesting that the changes in [^11^C]metoclopramide metabolism after cimetidine administration can be explained by an inhibition of CYP2D6 and CYP3A activity. Moreover, inhibition of CYP2D6 might be the cause for the trend towards a decrease in [^11^C]metoclopramide metabolism observed after the co-administration of a pharmacological dose of unlabeled metoclopramide (Table II), as metoclopramide has been shown to be not only a substrate but also a potent inhibitor of CYP2D6 (10, 11).

In addition to the pretreatment with prototypical cation inhibitors, the effect of OCT1/2 on the kidney and liver uptake of [^11^C]metoclopramide was investigated by examining wild-type and *Slc22a1/2*^*(−/−)*^ mice (40). The uptake rate constants of total radioactivity from blood into the kidney (*k*_uptake,kidney_) and from blood into liver (*k*_uptake,liver_) were significantly reduced in *Slc22a1/2*^*(−/−)*^ mice as compared to wild-type mice (Fig. 7). Kidney/plasma and liver/plasma concentration ratios calculated at 15 min after radiotracer injection indicated that both renal and hepatic uptake were reduced in transporter knockout animals (Fig. 8). Similar effects have been shown for other known OCT1/2 substrates, such as metformin and sulpiride, for which a decrease in kidney/plasma and liver/plasma concentration ratios was observed in *Slc22a1/2*^*(−/−)*^ mice as compared to wild-type mice (31, 32, 41). It is noteworthy that *Slc22a1/2* knockout mice showed no increases in the plasma concentrations of [^11^C]metoclopramide, while cimetidine significantly increased the plasma concentration of [^11^C]metoclopramide (Fig. 4). This can be most likely explained by an additional inhibition of metabolic clearance of [^11^C]metoclopramide (i.e. metabolism and urinary excretion of radiolabeled metabolites) by cimetidine.

Our results suggest that the cation transporters OCT1/2 and MATEs were involved in the disposition of [^11^C]metoclopramide in mice. Changes in [^11^C]metoclopramide pharmacokinetics were moderate after *Slc22a1/2* knockout, suggesting that renal and hepatic uptake by OCT1/2 are not the rate-limiting steps in [^11^C]metoclopramide clearance in mice. A considerable part of the kidney and liver uptake of [^11^C]metoclopramide probably occurs via additional, unidentified uptake carriers and/or passive diffusion. This is supported by the results of Hendrickx et al. (24), who found that cellular uptake of metoclopramide in human OCT1 or OCT2 transfected cell lines was only 45% and 24% related to OCT1 and OCT2 activity, respectively. We were not able to separately assess the effect of MATE inhibition alone and inhibition of metabolism alone on [^11^C]metoclopramide disposition to elucidate which of these two processes was responsible for the rate-limiting steps in [^11^C]metoclopramide clearance. A previous study has shown that [^11^C]metoclopramide is metabolized to a considerably lower extent in humans than in mice (23). Approximately 65% of total radioactivity in human plasma was in the form of unchanged [^11^C]metoclopramide at 20 min after i.v. injection of a microdose of [^11^C]metoclopramide (23), while only 18% of total radioactivity in mouse plasma was in the form of unchanged [^11^C]metoclopramide at 15 min after injection in untreated wild-type mice (see Table II), which could mean that cation transporters may play a potentially greater role in the clearance of [^11^C]metoclopramide in humans than in mice.

Although caution is warranted when extrapolating rodent data to humans, our data suggest that alongside the saturation of metabolizing enzymes, saturation of OCT and MATE activity may at least partially contribute to the previously reported non-linearity in metoclopramide pharmacokinetics in rats and humans (9, 14, 15, 17). Moreover, our data suggest that metoclopramide may be a potential victim of metabolic or transport drug-drug interactions, although it should be noted that the investigated inhibitors were employed in the present work at far greater doses than those used in the clinic. Changes in enzyme or transporter activities caused by drug-drug interactions, disease or genetic polymorphisms may lead to changes in metoclopramide plasma pharmacokinetics, which may in turn affect the brain exposure to metoclopramide (Fig. 9) and thereby influence the occurrence and severity of its central side effects. This is supported by a previous study which established an association between polymorphisms in the *CYP2D6* gene and the occurrence of central side effects of metoclopramide (6).

## Conclusion

We were able to show that kidney and liver uptake of [^11^C]metoclopramide in mice is partially mediated by transport by OCT1 and OCT2, while MATE transporters contributed to the urinary excretion of [^11^C]metoclopramide and its radiolabeled metabolites. While transport by OCT1/2 was apparently not the rate-limiting step in [^11^C]metoclopramide clearance in mice, it cannot be ruled out that alterations in OCT1/2 and MATE activities, alongside the alterations in the activity of metabolizing enzymes, may also contribute to variability in metoclopramide plasma and tissue exposure and thereby cause variability in the side effects and clinical response to metoclopramide. Our study highlights the importance of considering the contribution of radiolabeled metabolites to the PET signal for assessing transporter effects in cases in which significant drug metabolism occurs over the duration of the PET scan. We provide a practical set-up in which the combination of PET imaging with metabolism experiments allows transporter effects to be differentiated from metabolism effects.

### Acknowledgements and Disclosures

The authors wish to thank Mathilde Löbsch for help in conducting the PET experiments. This work was supported by the Austrian Science Fund (FWF) [grant KLI 694-B30, to O.L.].

## Abbreviations

AUC: Area under the concentration-time curve
i.v.: Intravenous
MATEs: Multidrug and toxin extrusion proteins
OCTs: Organic cation transporters
P-gp: P-glycoprotein
PET: Positron emission tomography
TLC: Thin-layer chromatography
